# Identification of insulin-sensitizing molecules acting by disrupting the interaction between the Insulin Receptor and Grb14

**DOI:** 10.1038/s41598-017-17122-6

**Published:** 2017-12-04

**Authors:** Anaïs Gondoin, Cornelia Hampe, Richard Eudes, Cyril Fayolle, Cécile Pierre-Eugène, Maria Miteva, Bruno O. Villoutreix, Florence Charnay-Pouget, David J. Aitken, Tarik Issad, Anne-Françoise Burnol

**Affiliations:** 1 Institut Cochin, Université Paris Descartes, CNRS (UMR8104), Paris, France; 2 INSERM, U1016 Paris, France; 3 Université Paris Diderot, Sorbonne-Paris-Cité, Inserm UMR-S 973, Molécules Thérapeutiques in silico, Paris, France; 4 CP3A Organic Synthesis Group, ICMMO, UMR 8182, CNRS, Université Paris Sud, Université Paris Saclay, Orsay, France

## Abstract

Metabolic diseases are characterized by a decreased action of insulin. During the course of the disease, usual treatments frequently fail and patients are finally submitted to insulinotherapy. There is thus a need for innovative therapeutic strategies to improve insulin action. Growth factor receptor-bound protein 14 (Grb14) is a molecular adapter that specifically binds to the activated insulin receptor (IR) and inhibits its tyrosine kinase activity. Molecules disrupting Grb14-IR binding are therefore potential insulin-sensitizing agents. We used Structure-Based Virtual Ligand Screening to generate a list of 1000 molecules predicted to hinder Grb14-IR binding. Using an acellular bioluminescence resonance energy transfer (BRET) assay, we identified, out of these 1000 molecules, 3 compounds that inhibited Grb14-IR interaction. Their inhibitory effect on insulin-induced Grb14-IR interaction was confirmed in co-immunoprecipitation experiments. The more efficient molecule (C8) was further characterized. C8 increased downstream Ras-Raf and PI3-kinase insulin signaling, as shown by BRET experiments in living cells. Moreover, C8 regulated the expression of insulin target genes in mouse primary hepatocytes. These results indicate that C8, by reducing Grb14-IR interaction, increases insulin signalling. The use of C8 as a lead compound should allow for the development of new molecules of potential therapeutic interest for the treatment of diabetes.

## Introduction

Changes in lifestyle over the last decades resulted in a dramatic increase in the prevalence of metabolic diseases such as obesity and type 2 diabetes, which are characterized by an insulin-resistance state^1^. To overcome this defect, two different strategies are commonly used, aiming either at increasing insulin secretion or at improving distal insulin action, in order to decrease hepatic glucose production and enhance peripheral glucose utilization^2^. However, when the disease progresses, these treatments often become less efficient and type 2 diabetic patients need to resort to insulinotherapy. The identification of new molecular targets therefore remains an important issue for the development of innovative therapeutical approaches to improve insulin action^3^.

The first step of insulin action is its binding to its membrane receptor (IR), a member of the tyrosine kinase family of receptors, and the activation of intracellular pathways leading to the final anabolic effects of the hormone^4^. Binding of insulin to its receptor induces conformational changes that bring the two β-subunits nearer to each other, leading to trans-autophosphorylation of the β-subunits on tyrosine residues. The activated receptor then recruits and phosphorylates intracellular substrates such as IRSs and Shc, therefore initiating downstream signaling pathways (MAP kinases, PI3-Kinase) and promoting the physiological effects of insulin^5^. However, accurate regulation of insulin action results from a tight balance between activation of these signaling pathways and simultaneous induction of feedback mechanisms. These negative regulations involve the action of protein tyrosine phosphatases^6,7^ as well as adaptor proteins, like the Grb7 family of proteins^8,9^.

Adapters of the Grb7 family, which includes Grb7, Grb14, and Grb10, are involved in the regulation of tyrosine kinase receptor signaling^10^. Grb14 is selectively expressed in insulin-sensitive tissues and is rapidly recruited to the activated receptor upon insulin stimulation^11–13^. Grb14 binds mainly through its Between PH and SH2 (BPS) domain to the phosphorylated insulin receptor tyrosine kinase domain (IRTK) and acts as a pseudosubstrate to inhibit its catalytic activity^11,14,15^. Interestingly, recent genetic studies suggested an association between the locus of Grb14 and diabetes, insulin resistance or fat distribution^16–18^. Moreover, Grb14 expression is increased in adipose tissue of insulin-resistant animal models and type 2 diabetic patients, and improvement of insulin sensitivity in ob/ob mice by treatment with an antidiabetic drug (thiazolidinedione) is associated with a reduction in Grb14 expression^19^. Grb14 expression is also increased in skeletal muscle of obese and insulin-resistant patients and decreased by weight loss, along with increased insulin sensitivity^20^. All these data suggest that this protein could be involved in the decrease in insulin signaling observed in metabolic diseases. Targeting IR-Grb14 interaction, in order to relieve the Grb14 inhibitory action on the IR, may thus represent a new strategy to improve insulin signaling and action.

To identify small non-peptidic inhibitors of the IR-Grb14 interaction, we performed structure-based virtual ligand screening experiments, based on the Grb14(BPS)-IR crystallographic structure^15,21–24^. After a structural analysis of docked molecules present in the ChemBridge database in two different pockets at the surface of the IR, we selected a list of 1000 molecules that were experimentally tested for disrupting the Grb14-IR interaction in an a-cellular BRET (Bioluminescence resonance Energy Transfer) assay^25,26^. Three compounds were isolated and further characterized for their activity on insulin signaling.

## Results and Discussion

### Virtual screening and high-throughput screening

**Figure 1:**
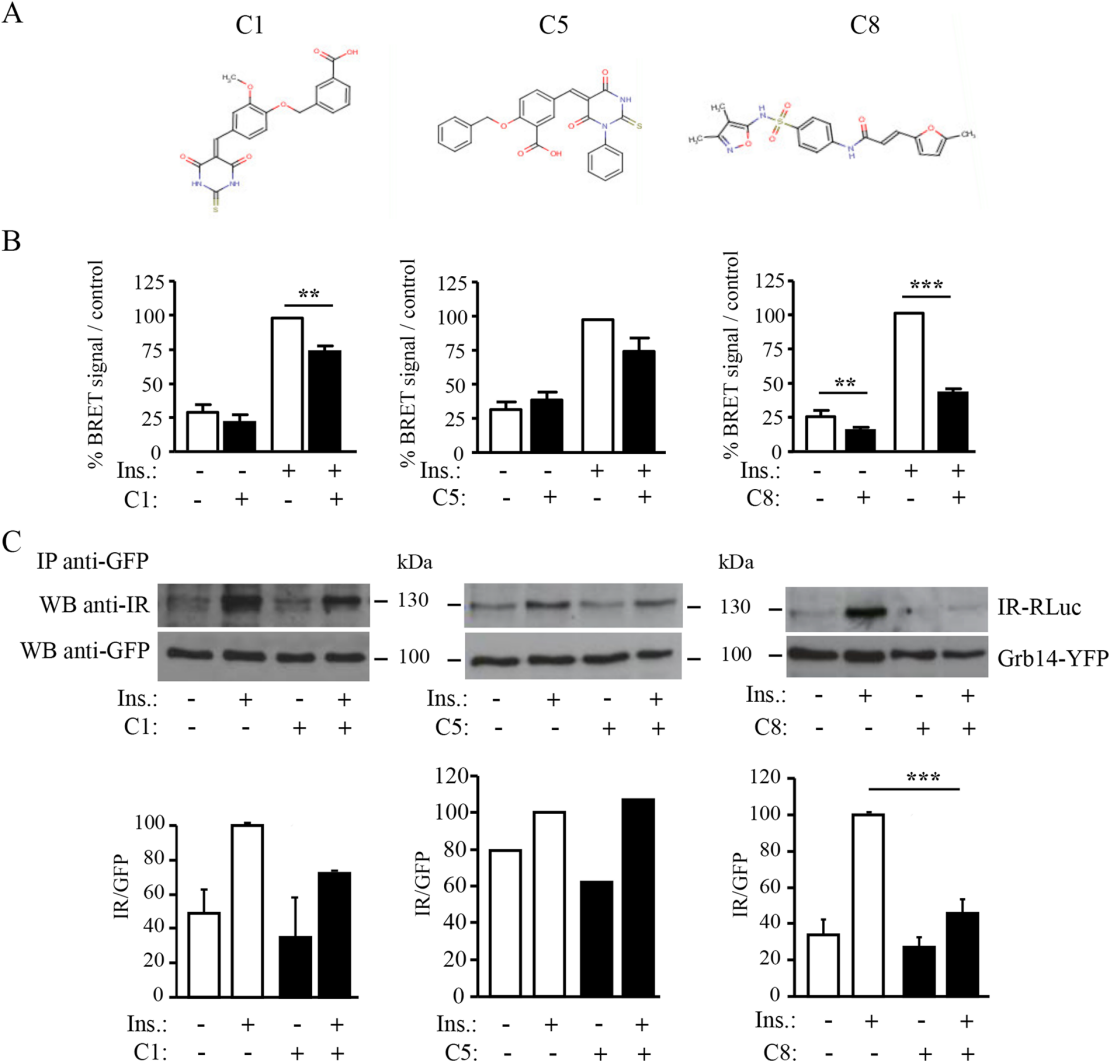
Effect of compounds C1, C5 and C8 on IR-Grb14 interaction. (**A**) Molecular structure of compounds C1, C5 and C8. (**B**) BRET assays using partially purified IR-RLuc and Grb14-YFP fusion proteins were performed *in vitro* in the presence of 50 µM of compounds as described in *Methods*. Results are the means ± SEM of 3 (C1, C5) to 8 (C8) independent experiments and are expressed as percent of BRET signal obtained with insulin-stimulated receptors incubated with control DMSO. (**C**) IR-Rluc and Grb14-YFP were incubated as in (**B**). Immunoprecipitation was then performed using an anti-GFP antibody. The amount of IR-RLuc co-precipitated with Grb14-YFP was evaluated by immunoblotting using an anti-IR antibody. Histograms, expressed as percent of control, represent the means ± SEM of the densitometric quantification of 2 (C1, C5) to 8 (C8) independent experiments (**p < 0.01, ***p < 0.001, statistical analysis was performed using ANOVA followed by Bonferroni’s Multiple Comparison Test).

We have scored a collection containing 340 000 compounds (ChemBridge) after structured-based virtual screening performed on the basis of the Grb14(BPS)-IR crystallographic structure^15^. Two binding pockets were identified and used for screening (Supplementary Fig. 1). After structural analysis of the protein-ligand complexes and investigation of the top scores, a list of molecules expected to bind to pocket 1 (relatively long binding groove with one very polar and positively charged side while the other end is essentially hydrophobic and aromatic) or to pocket 2 (relatively small and mainly hydrophobic cavity) was selected. At the end of the *in silico* analysis, a list of 1000 compounds were purchased and experimentally evaluated for inhibition of IR-Grb14 interaction using the a-cellular BRET assay as described in *Methods*. Three molecules (Fig. 1A), expected to bind to pocket 1, were reproducibly detected in 3 independent screens as having inhibitory activity on BRET between IR-luc and Grb14-YFP. The effects of these compounds (C1, C5 and C8) were then further studied in BRET (Fig. 1B) and co-immunoprecipitation (Fig. 1C) experiments. BRET signal between IR and Grb14 was 3 to 4 times higher when measured with purified receptors from insulin-stimulated HEK-293T cells compared to purified receptors from non-stimulated cells, indicating that binding of Grb14 to the IR was indeed dependent upon IR activation, as demonstrated previously using different experimental settings^11,14,27^. Used at 50 µM, the three compounds C1, C5 and C8 decreased the BRET signal between the activated insulin receptor and Grb14 respectively by 25%, 18% (not significant) and 60%. Of note, C8 also induced a significant decrease (50%) in the BRET signal in the absence of insulin stimulation. In co-immunoprecipitation experiments, as expected, the association between IR and Grb14 was also much more prominent with purified receptors from insulin-stimulated HEK-293T cells compared to purified receptors from non-stimulated cells. C1 and C8 reduced respectively by 30% and 60% the amount of IR co-precipitated with Grb14, while C5 had no effect. C8 was therefore the most efficient compound to inhibit insulin-induced IR-Grb14 interaction, both in BRET and co-IP experiments.

### Inhibitory activity of compounds with a structure similar to C1, C5 or C8

**Figure 2:**
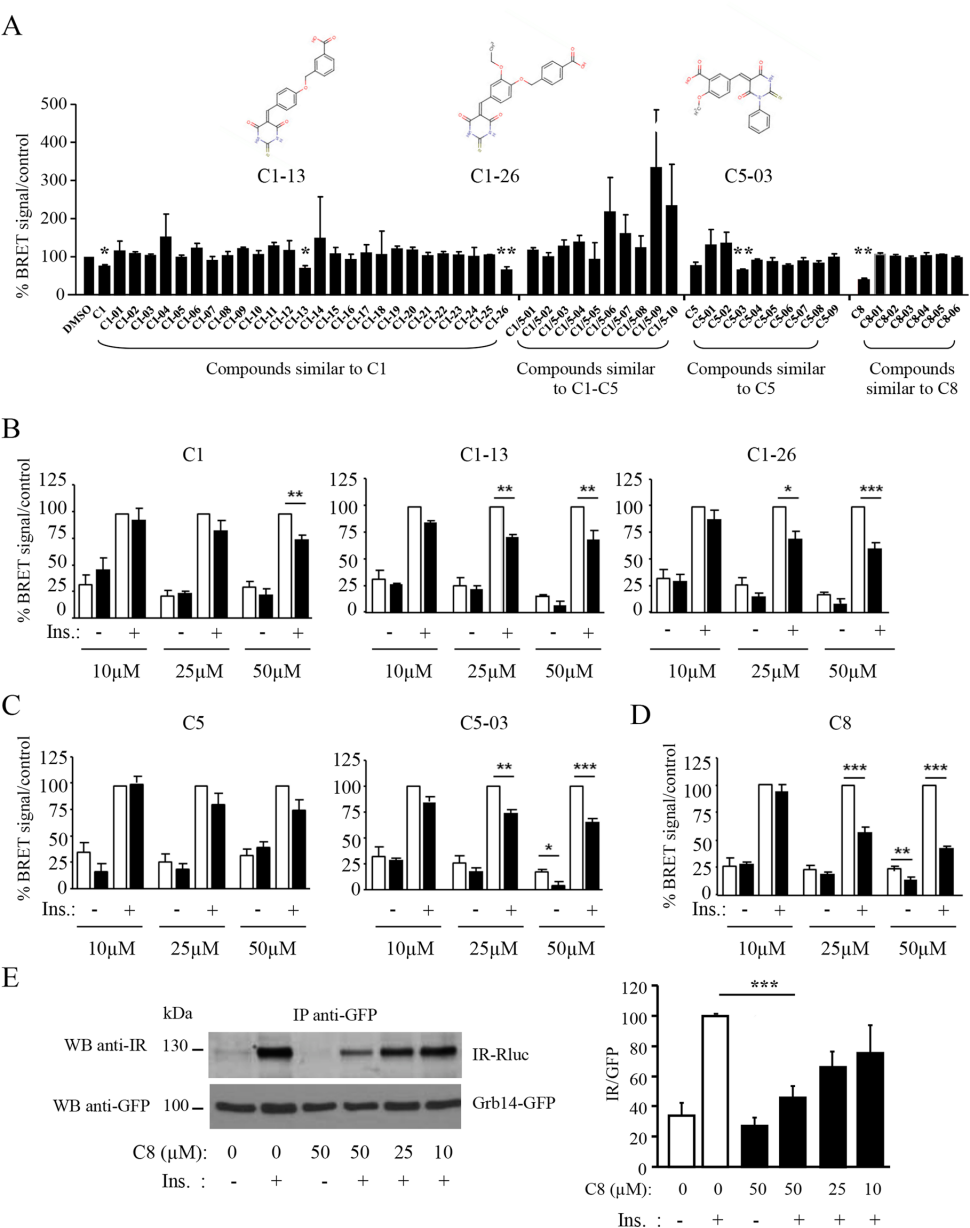
Effect of derivated-compounds on IR-Grb14 BRET interaction. (**A**) BRET assays were performed in the presence of 50 µM of compounds. Results, expressed as percent of the control DMSO, are the means ± SEM of 3 to 8 independent experiments (*p < 0.01, **p < 0.001 when compared to DMSO, using ANOVA followed by Dunnett Multiple Comparison tests). The structure of the compounds C1-13, C1-26 and C5-03 identified in this assay is shown in the upper part of the panel. (**B**–**D**) Dose-response experiments using the IR-Grb14 BRET system. Open bars: DMSO control, black bars: compounds added at the indicated concentrations. Results are the means ± SEM of 3 to 8 independent experiments and are expressed as percent of control. (**E**) Dose-response experiments using anti-GFP immunoprecipitation. Histograms represent the means ± SEM of the densitometric quantification of 3 to 6 independent experiments and are expressed as percent of the value measured in the presence of insulin (*p < 0.05, **p < 0.01, ***p < 0.001, using ANOVA followed by Bonferroni’s Multiple Comparison Test for **B**–**E**).

In an attempt to identify compounds with improved inhibitory properties, similarity search was performed on the basis of C1, C5 and C8 structures. The ChemBridge database (containing 4,000,000 molecules) was scanned for compounds with shape similar to C1, C5, or C8, or compounds similar to both C1 and C5. Using structured-based and ligand-based virtual screening and different virtual filters, 51 molecules with high-score for potential inhibition of the IR-Grb14 interaction were selected, purchased and experimentally evaluated for inhibition of IR-Grb14 interaction using the a-cellular BRET assay (Fig. 2A). This group contained all compounds similar to C1, C5 and C8, and 10 compounds similar to both C1 and C5 exhibiting the best properties. Some compounds displayed artifactual increase in BRET signal, due to direct effects on luciferase activity (C1-04, C1-14. C1/5-06, C1/5-07, C1/5-09, C1/5-10). Three new molecules (C1-13 and C1-26 similar to C1, and C5-03 similar to C5) were reproducibly detected as having inhibitory activity on BRET between IR and Grb14 (Fig. 2A), but none of the compounds similar to C8 had any effect. Dose-response experiments confirmed the 25% reduction of IR-Grb14 BRET signal by C1 at 50 µM, but lower doses of this compound did not appear to be effective. However, C1-13 and C1-26 (C1-derived compounds) were active at 25 µM (significant reduction of 30% of BRET signal) (Fig. 2B). C5-03 (C5-derived compound) also induced a significant 25% decrease of IR-Grb14 BRET signal at 25 µM and at 50 µM decreased both basal and insulin-induced BRET signal (Fig. 2C). C8 decreased BRET signal between IR and Grb14 by 50% at a concentration of 25 µM (Fig. 2D). Co-immunoprecipitation experiments also showed dose-dependent inhibition by C8 of the amount of IR immunoprecipitated with Grb14, with a reduction of 20% at a concentration of 25 µM and 50% at a concentration of 50 µM (Fig. 2E).

Thus, from our initial screen and the following search for compounds with structural similarity with the 3 initial hits, we identified 5 compounds effective in decreasing the IR-Grb14 interaction. C8 remained the most active, as we did not succeed to find a more active compound among C1, C5 and C8-derived compounds. Furthermore, *in silico* analysis of the molecules C1 and C5 (and molecules similar to these two compounds) suggested that under some experimental conditions these molecules could interfere with some assays^28^. In this context, we decided thereafter to focus on the investigation of the inhibitory activity of C8.

### Characterization of C8 inhibitory activity

**Figure 3:**
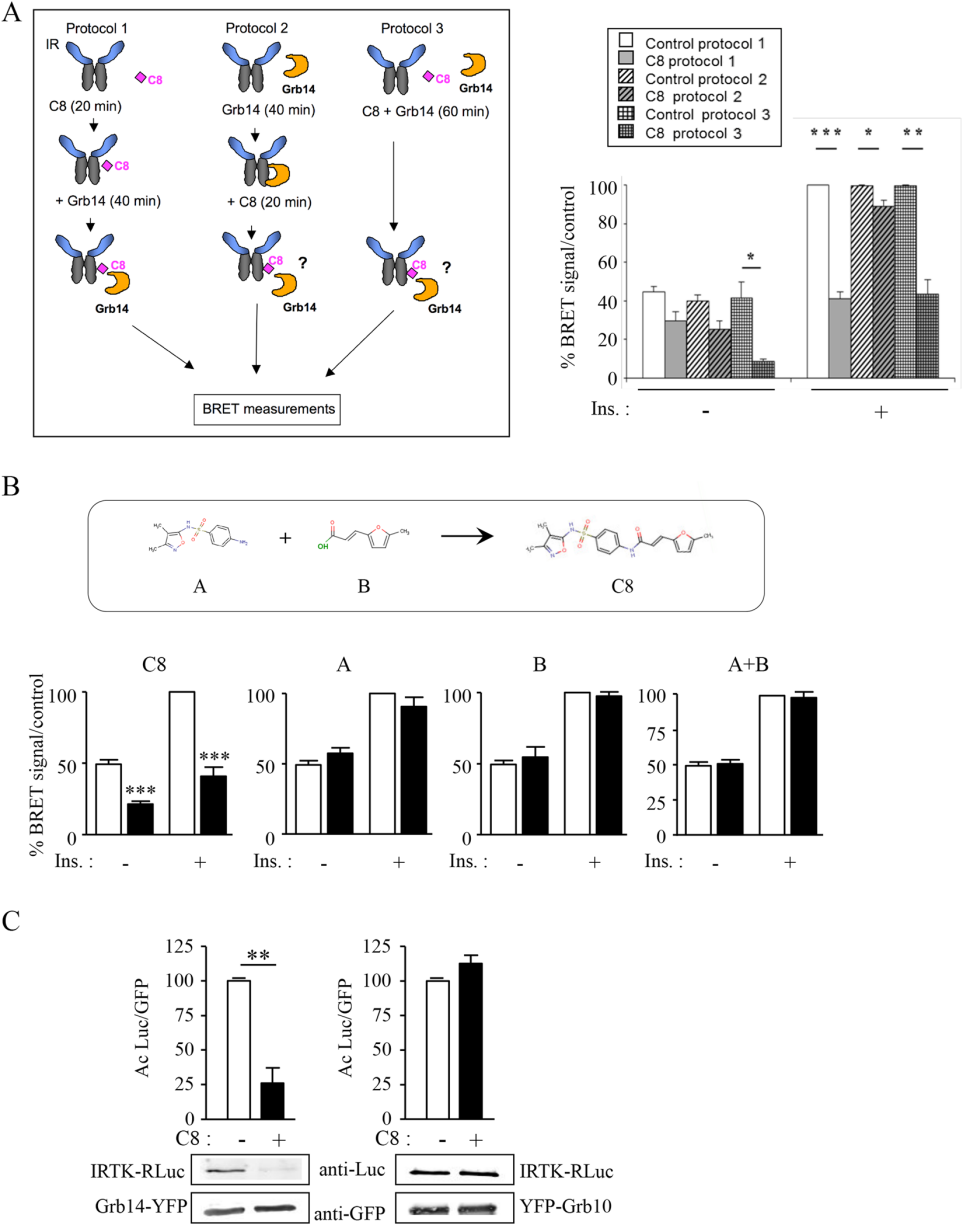
Characterization of C8 inhibitory activity. (**A**) Left part: schematic representation of the three BRET protocols used in this experiment. Right part: Effect of C8 (50 µM) on IR-Grb14 BRET signal in each of the three protocols. Results, expressed as percent of BRET signal obtained with insulin-stimulated receptors incubated with control DMSO, are the means ± SEM of 4 independent experiments (*p < 0.05, **p < 0.01, ***p < 0.001, using ANOVA followed by Bonferroni’s Multiple Comparison Test). (**B**) Effect of A and B, the two intermediate compounds involved in the last step of C8 synthesis, on the IR-RLuc/Grb14-YFP interaction measured by BRET *in vitro*. Results, expressed as percent of BRET signal obtained with insulin-stimulated receptors incubated with control DMSO, are the means ± SEM of 3 to 4 independent experiments (***p < 0.001, using ANOVA followed by Bonferroni’s Multiple Comparison Test). (**C**) Co-immunoprecipitation of the IRTK with Grb14 or Grb10. Cells were co-transfected with either IRTK48-Rluc and Grb14-YFP (left part), or IRTK48-Rluc and YFP-Grb10 (right part), and incubated in the absence or the presence of 50 µM of compound C8 as indicated. Immunoprecipitation was performed using an anti-GFP antibody, and the amount of IRTK-RLuc co-precipitated with Grb14-YFP or YFP-Grb10 was evaluated by immunoblotting using an anti-Luc antibody. Histograms represent the means ± SEM of the densitometric quantification of 3 independent experiments. Results are expressed as percent of control (**p < 0.01, t-test).

In the initial screen the compounds were incubated with IR before adding Grb14, implying that they acted by preventing the association between the two proteins (Protocol 1). To check this model, two other BRET protocols were tested in the same experiment (Fig. 3A). In “Protocol 2”, extracts from Grb14-YFP transfected cells were preincubated with partially purified IR-Luc for 40 minutes before the addition of C8. This protocol was designed to investigate a potential displacement of the IR-Grb14 interaction by C8. In “Protocol 3” Grb14-YFP fusion proteins, partially purified IR-Luc and C8 were added simultaneously, to test a possible competition between C8 and Grb14 for IR binding. The C8 inhibitory effect was greatly reduced when Grb14 was pre-bound to the activated receptor, with a decrease in the IR-Grb14 BRET signal by only 10% (Protocol 2). However, when the Grb14 and C8 were added simultaneously to the partially purified IR (Protocol 3), C8-induced decrease in IR-Grb14 BRET signal was similar to the effect measured in Protocol 1 (60% inhibition) (Fig. 3A). These results showed that although C8 was much less efficient to displace Grb14-IR interaction when Grb14 was already bound to the receptor, it did not need to be preincubated with the IR to impede Grb14 binding.

To investigate whether a partial structure of C8 retains inhibitory activity on IR-Grb14 interaction, we investigated separately the activity of the two molecules that are involved in the last step of the synthesis of the compound. The synthesis of C8 was achieved from commercially available sulfafurazole antibiotic (named “compound A”) and 5-methyl-2-furfuraldehyde in two steps. The first step was the conversion of 5-methyl-2-furfuraldehyde into (*E*)-3-(5-methyl-2-furanyl)-2-propenoic acid (named “compound B”). The second step consisted in the condensation of the antibiotic with the acid in the presence of a peptide coupling reagent (Fig. 3B). We tested the activity of these intermediate compounds using the *in vitro* IR-Grb14 BRET assay. In this series of experiments, C8 displayed the same inhibitory effect as previously described, but the intermediate compounds, added either separately or together, had no effect on IR-Grb14 interaction (Fig. 3B). This suggests that the inhibitory activity of C8 can not be obtained with a partial structure of the compound and that the whole molecule is required.

The virtual docking that permitted the identification of C8 was performed using the crystal structure of the Grb14 BPS region in complex with the IR kinase domain^15^. To confirm that C8 reduced IR-Grb14 interaction by acting specifically on the kinase domain of the insulin receptor (IRTK), immunoprecipitations were performed in cells co-expressing the IRTK-Rluc and the Grb14-YFP chimeric proteins and which were treated or not with C8 (Fig. 3C). IR α-subunit exerts a permanent break on the kinase activity and, when expressed alone, the tyrosine kinase domain is constitutively activated^29^. As shown in Fig. 3C, the addition of C8 induced a 75% decrease in the amount of IRTK co-immunoprecipitated with Grb14. This result indicates that C8 enters into the cells and reduces the interaction between Grb14 and IRTK (Fig. 3C left part). To test the specificity of this effect of C8, similar experiments were performed using the Grb10 protein. Grb10 which is structurally closely related to Grb14, also binds to the activated IR and inhibits its catalytic activity^14,30^ but may not involve the exact same type of interactions as compared to Grb14 and IRTK^14,15^. Grb10 co-precipitated with IRTK, but the amount of IRTK co-immunoprecipitated with Grb10 was not altered by the addition of C8 (Fig. 3C right part). The inhibitory activity of C8 thus seems specific for the IR-Grb14 interaction, hence suggesting that C8 should bind in a region of IRTK that is less important for the IR-Grb10 interaction.

### Evaluation of C8 toxicity

C8 cytotoxicity was investigated on growth and survival in HEK-293T cells using the UptiBlue assay, which permits to follow the growth of the cell population in the same well during several days^31^. Cells were cultured for 72 hours in the presence of different serum concentrations (0.1%, 1%, and 10%), in the absence or the presence of C8 (50 or 100 µM). A 7-fold increase in the cell population was observed when cells were cultured with 0.1% serum and a 10-fold increase was observed with 1% or 10% serum. The addition of C8 did not alter cell growth both at low- and high-serum concentration (Supplementary Fig. 2).

### Effect of C8 on insulin receptor activation

**Figure 4:**
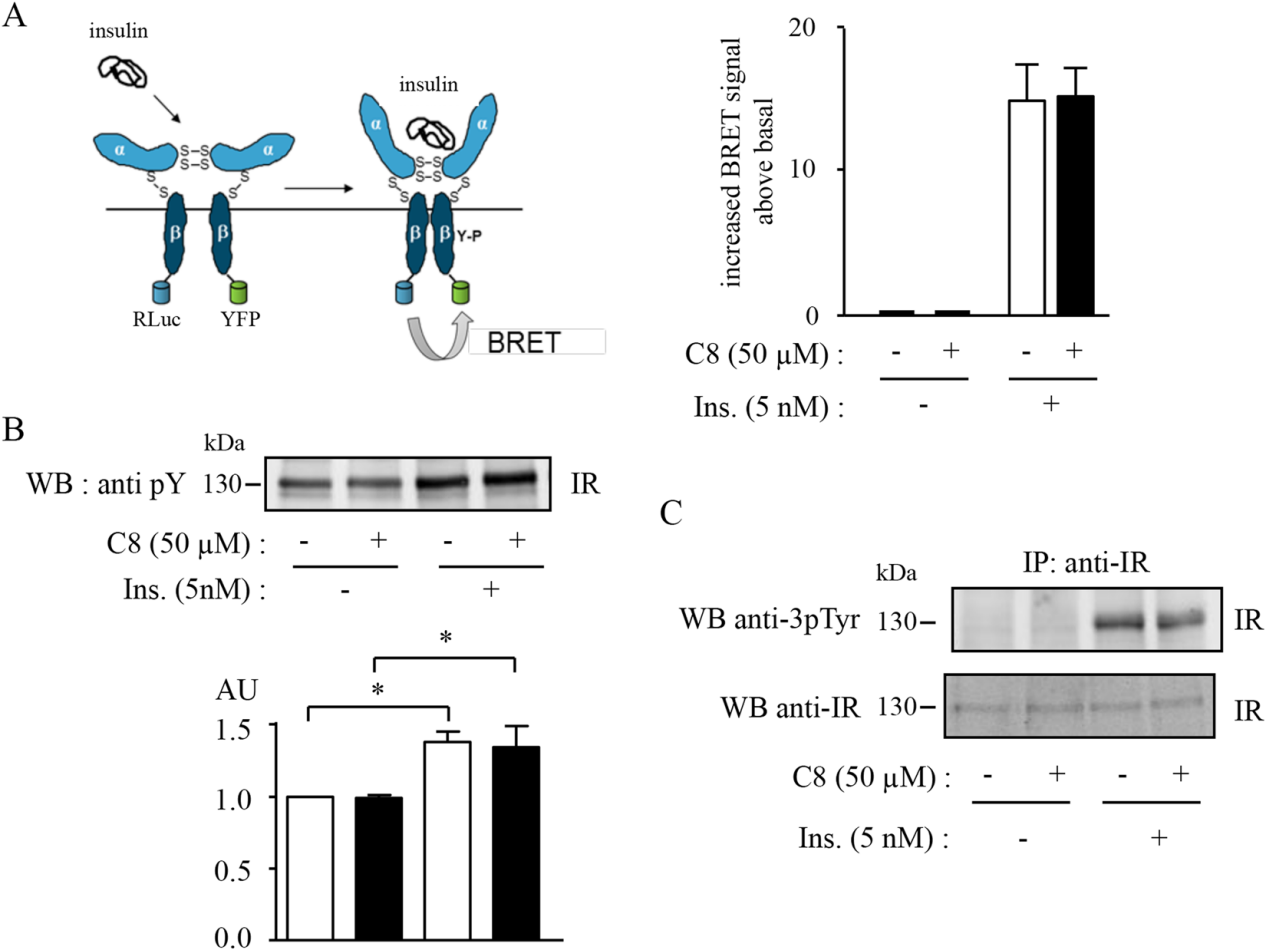
C8 did not directly activate the IR. (**A**) Left panel: schematic representation of the IR conformational change induced by insulin. Right panel: BRET assays were performed *in vitro* in the absence or in the presence of 5 nM insulin and 50 µM of compound C8 as described in *Methods.* Results (increase BRET signal above basal) are the means ± SEM of 3 independent experiments. (**B**) Autophosphorylation of partially purified fusion receptors was performed as described in *Methods* in the absence or the presence of 50 µM C8 and 5 nM insulin. Autophosphorylation of the receptor-fusion protein was detected by immunoblotting using an antiphosphotyrosine antibody. Histograms represent the means ± SEM of the densitometric quantification of 3 independent experiments (*p < 0.05, using ANOVA followed by Newman-Keuls Multiple Comparison Test). (**C**) HEK-293T cells were lysed and endogenous receptors were immunoprecipitated (IP) with an anti-IR antibody and protein G-sepharose beads. Autophosphorylation was detected by immunoblotting using an antibody directed against the three phosphotyrosines of the kinase domain.

As the initial virtual screening was designed to identify molecules interacting with the IR β-subunit, it cannot be excluded that C8 may directly affect IR conformation and activity. To evaluate whether C8 can induce conformational changes within the IR we used a BRET-based method previously developed to monitor ligand-induced conformational changes within the IR dimer (Fig. 4A left panel)^32^. In the absence of insulin, a basal BRET signal occurs, reflecting random interactions between Rluc and EYFP (15.6 ± 1.9 mBU). C8 had no effect on basal BRET (17.3 ± 2.4 mBU). As shown previously, binding of insulin to its receptor stimulated BRET signal. The addition of C8 did not alter this insulin-induced BRET signal, suggesting that the conformational changes induced by insulin binding were not modified in the presence of C8 (Fig. 4A right panel). The effect of C8 on IR activation was further investigated in Western blotting experiments. *In vitro* insulin-induced autophosphorylation of partially purified IR was not altered by the addition of C8 (Fig. 4B). Similarly, the autophosphorylation of endogenous IR in intact cells was not modified by the addition of C8 (Fig. 4C). Altogether, these data show that C8 did not affect insulin-induced conformational changes within the receptor and did not modify IR autophosphorylation. Therefore, potential effects of C8 on IR signaling are unlikely to be due to direct activation of the receptor.

### Effect of C8 on insulin signaling using BRET in living cells

Relieving Grb14 inhibitory action on the IR by C8 is expected to induce an increase in insulin signaling pathways. We thus used different cellular BRET assays to study the consequences of C8 addition on the activation of MAP kinase and PI3-kinase signaling pathways.

**Figure 5:**
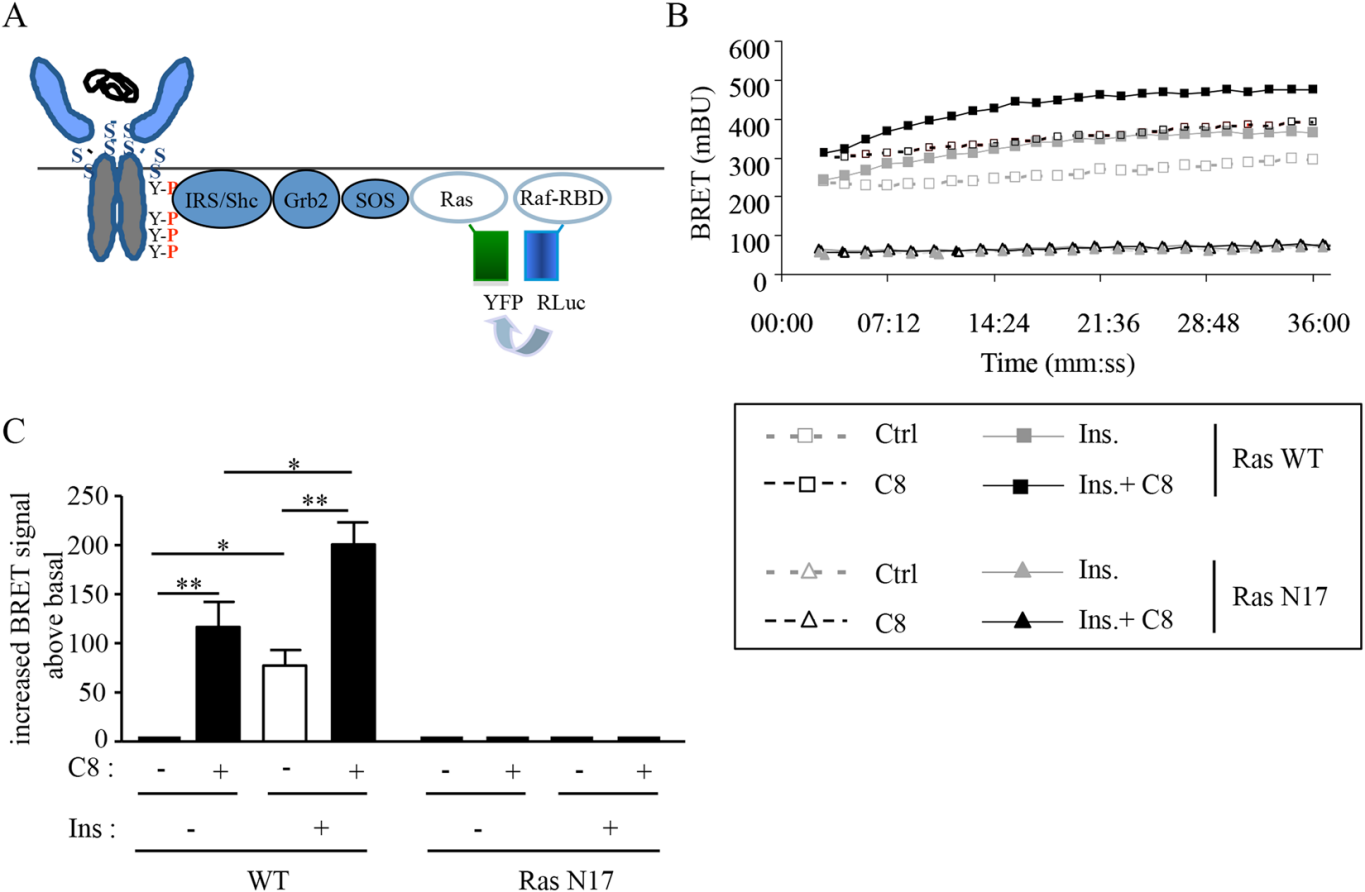
Activation of the Ras/Raf pathway by C8 in intact cells. (**A**) Schematic representation of the BRET assay used in this experiment. (**B**) Typical experiment showing BRET signal monitored during 36 min in HEK-293T cells co-expressing the Ras Binding Domain of Raf fused to the *Renilla* luciferase (Raf-RBD-RLuc) and either WT or mutated pEYFP-RasN17. Cells were stimulated or not with 5 nM of insulin and compound C8 (50 µM) as indicated. (**C**) Quantification of insulin-induced BRET (increase BRET above basal). Results are the means ± SEM of 4 independent experiments (*p < 0.05, **p < 0.01, using ANOVA followed by Newman-Keuls Multiple Comparison test).

A BRET assay monitoring the Ras-Raf interaction in living cells was developed (Fig. 5A). In the absence of insulin, a stable basal BRET signal could be detected, and the addition of insulin rapidly increased this signal. Attesting the specificity of this assay, insulin had no effect on the BRET signal when a dominant negative mutant of Ras (pEYFP-RasN17) was used (Fig. 5B,C). The addition of C8 significantly increased the Ras-Raf interaction measured in the absence and in the presence of 5 nM insulin (Fig. 5B,C).

**Figure 6:**
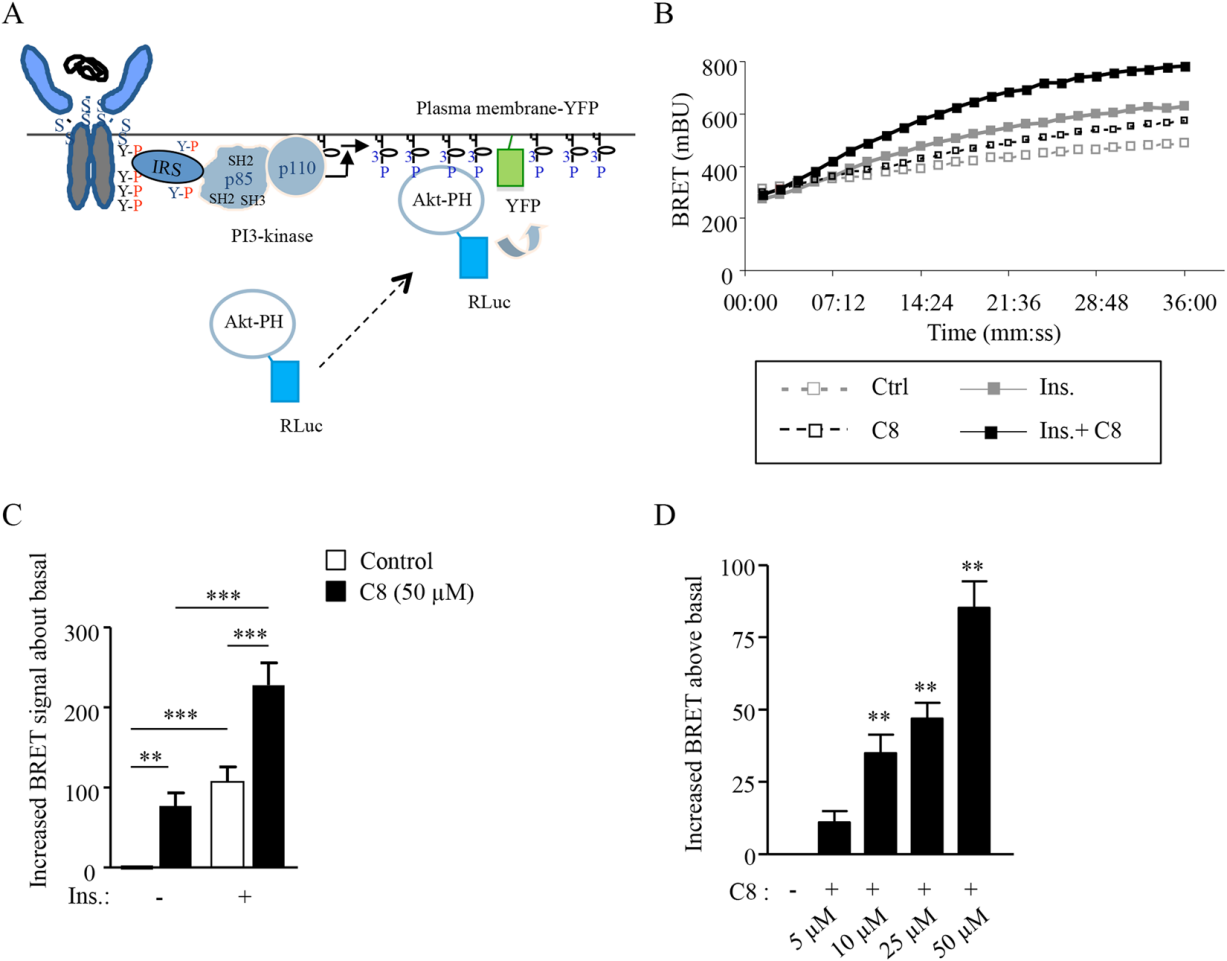
Activation of the PI3K/Akt pathway by C8 in intact cells. (**A**) Schematic representation of the BRET assay used in this experiment. (**B**) Typical experiment showing real-time insulin and C8 effects on PIP_3_ production in HEK-293T cells. (**C**) Insulin-induced BRET and effect of C8 on the translocation of Akt to the plasma membrane (increase BRET mBU above basal). (**D**) Dose-dependent effect of compound C8 on PIP_3_ production (increase BRET mBU above basal). Results are the means ± SEM of 3 to 8 independent experiments (**p < 0.01, ***p < 0.001, using ANOVA followed by Newman-Keuls (**C**) or Dunnett Multiple Comparison tests (**D**)).

To investigate the activation of the PI3K/Akt pathway, we recently developed a BRET assay allowing to measure the recruitment of the PH domain of Akt (Akt-PH) to the plasma membrane in response to PI-3 kinase-induced phosphatidylinositol-3,4,5 triphosphate (PIP_3_) production (Fig. 6A)^33^. As shown in Fig. 6B insulin rapidly increased the BRET signal in this assay, and the addition of C8 significantly increased the recruitment of Akt to the membrane in the absence and in the presence of 5 nM insulin (Fig. 6C). A dose-response experiment using increasing concentrations of C8 showed that an increase in PIP_3_ production was detectable at 5 µM of C8, and a significant increase in BRET signal was observed at 10 µM (Fig. 6D).

IR phosphorylation, and subsequent signaling activity, is tightly controlled by the protein tyrosine phosphatase 1B (PTP1B), which binds to activated IR and dephosphorylates the tyrosine residues present in the activation loop^6,7^. We verified whether C8 could interfere with IR-PTP1B complex formation. The IR-PTP1B interaction was measured by BRET as described previously^6^. We observed that the addition of C8 did not modify basal or insulin-induced IR-PTP1B complex formation (Supplementary Fig. 3). These results, together with the absence of modification of IR phosphorylation shown in Fig. 4, indicate that the improved activation of Ras and Akt signaling induced by C8 are unlikely to be attributed to modification of PTP1B activity.

The initial virtual docking of the chemical library was performed on a pocket present only in the structure of the activated IR, implying that the identified compounds should not bind to the non-phosphorylated IR in the basal state. Therefore, it may be intriguing that C8 compound increased basal BRET signal in Ras-Raf and PIP3 assays. However, it must be kept in mind that the IR consistently oscillates between active and inactive conformations, being predominantly in the inactive conformation in the absence of ligand and in the active conformation under insulin stimulation. It has been estimated that about 5–10% of the receptors are in the activated form in the absence of insulin^34^. Oscillation of the IR between inactive and active forms was clearly demonstrated by experiments showing that treatment of cells with vanadate, a protein tyrosine-phosphatase inhibitor, led to a significant increase in IR phosphorylation and in its tyrosine kinase activity, even in the absence of insulin^35,36^. Thus, in the absence of hormonal stimulation the IR undergoes cycles of phosphorylation/dephosphorylation, allowing Grb14 to bind when it is in its active conformation. The compound C8 could therefore inhibit a basal interaction between the IR and Grb14, explaining its activity observed in the absence of insulin using various experimental systems (Figs 1B, 2D, 5B and 6B–D).

### Effect of C8 on downstream biological effects of insulin

**Figure 7:**
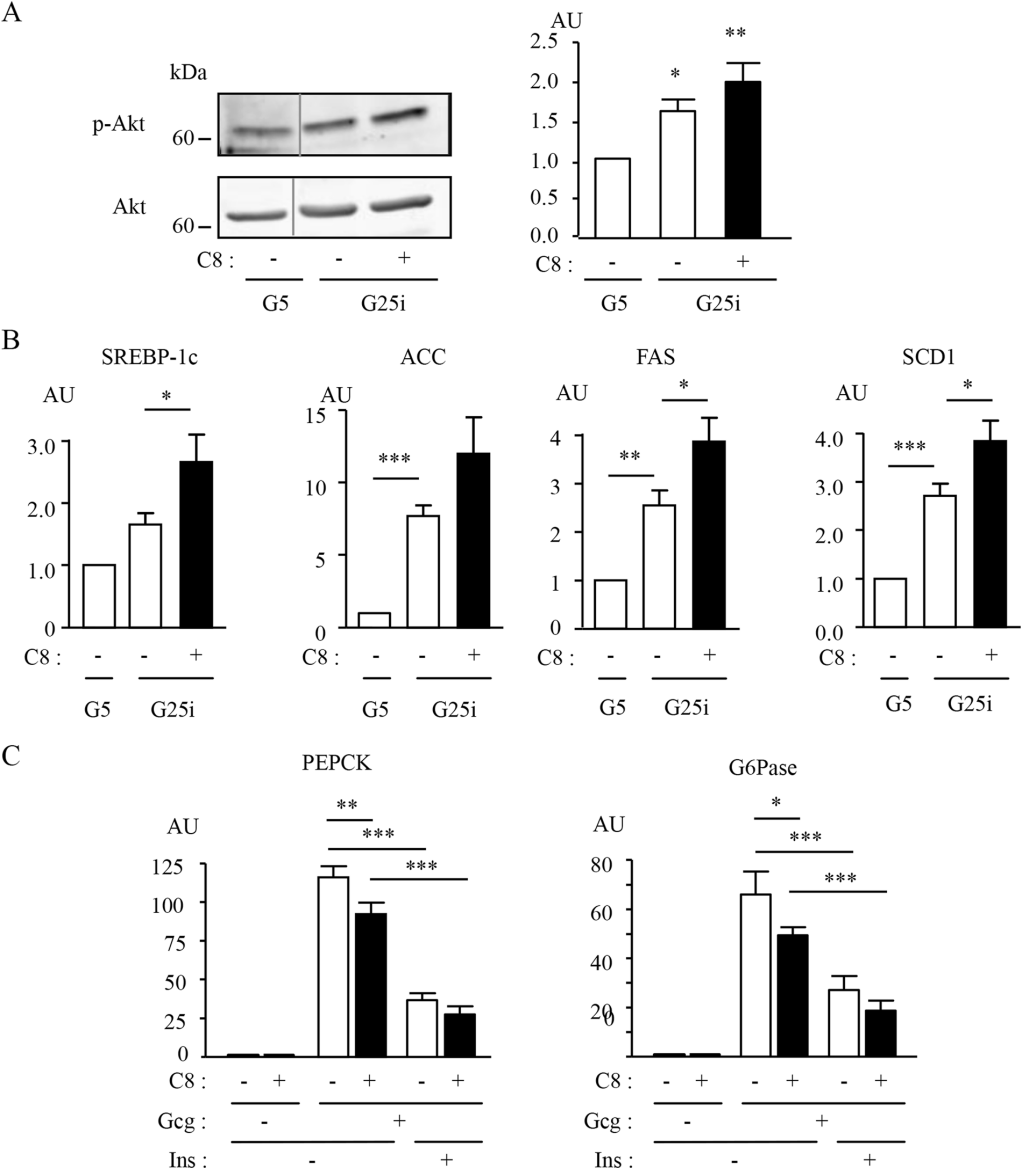
Effect of C8 on downstream biological effects of insulin. (**A**) Effect of C8 on Akt phosphorylation in primary hepatocytes. Primary mouse hepatocytes were cultured for 24 h in the presence of 5 mM glucose (G5) or 25 mM glucose and 10 nM insulin (G25i), with or without 50 µM of compound C8 as indicated. Cell lysates were analyzed by Western blotting using the indicated antibodies. The grey lines indicate a cropped lane of the original blots. The uncropped blots are shown in Supplementary Fig. 5. Histograms represent the means ± SEM of the densitometric quantification of 3 independent experiments. (**B**) Expression of lipogenic genes. Primary mouse hepatocytes were cultured for 24 h in the presence of G5 or G25i with or without 50 µM of compound C8. mRNA expression level was measured by qRT-PCR. Results are normalized to the expression of 18S mRNA and correspond to the means ± SEM of 3 to 5 independent experiments. (**C**) Expression of gluconeogenic insulin target genes. Primary hepatocytes were preincubated 1 h in the presence of 5 mM glucose with or without 10 nM glucagon and treated for 8 h in the absence or presence of 1 nM insulin and 50 µM of compound C8. mRNA expression was measured as in (**B**). Statistical analysis was performed using ANOVA followed by Dunnett (**A**) or Newman-Keuls Multiple Comparison tests (**B**,**C**) (*p < 0.05, **p < 0.01, ***p < 0.001) AU: arbitrary units.

The consequences of the addition of C8 on downstream biological effects of insulin were then investigated in primary mouse hepatocytes, a physiological target of insulin. Primary mouse hepatocytes were cultured for 24 hours under low (G5) or high glucose plus insulin (G25i) concentrations, mimicking respectively the fasted and fed states, with or without C8. We first investigated the effect of C8 on insulin-induced activation of Akt, the major player in the transmission of insulin metabolic effects^4^. Akt phosphorylation was enhanced in G25i compared to G5 condition, as expected, but the addition of C8 led to a further increase in Akt phosphorylation (Fig. 7A). The stimulatory effect of C8 on Akt phosphorylation was confirmed in the αML12 hepatocyte cell line (Supplementary Fig. 4). In the liver, insulin stimulates fatty acid synthesis and inhibits gluconeogenesis, mainly by acting at a transcriptional level. Hepatic lipogenesis is controlled by insulin-induced activation of the transcription factor SREBP-1c (Sterol Regulatory Element Binding Protein-1c), which activates the expression of lipogenic genes such as ACC (Acetyl-CoA Carboxylase), FAS (Fatty Acid Synthase) and SCD-1 (Stearoyl-CoA Desaturase-1)^37^. As expected, SREBP-1c, ACC, FAS and SCD-1 mRNA expression was induced under G25i condition when compared to G5 condition. Interestingly, C8 significantly potentiated insulin-stimulated gene expression, by approximately 60% (Fig. 7B). To study the insulin inhibitory effect on gluconeogenic genes expression, primary hepatocytes were pre-incubated with or without 10 nM glucagon and then treated for 8 h in the absence or in the presence of 1 nM insulin and 50 µM of compound C8. As expected, glucagon-induced expression of Phosphoenolpyruvate carboxykinase (PEPCK) and Glucose-6 phosphatase (G6Pase) mRNA was inhibited by insulin. The addition of C8 induced a significant decrease of about 20% of PEPCK and G6Pase mRNA expression. However, when added to insulin, C8 did not further decrease gluconeogenic gene expression, although an inhibitory trend could be observed (Fig. 7C). Altogether, our data show that C8 is able to improve hepatocyte metabolism, increasing insulin effect on lipogenic gene expression and decreasing glucagon-induced expression of gluconeogenic genes.

## Conclusion

Considering the epidemic expansion of obesity and type 2 diabetes in this beginning century, two pathologies associated with an insulin-resistance state, the identification of new molecules capable of improving insulin action is of considerable medical interest. In the present study, using a structure-based virtual screening to search for molecules blocking Grb14 binding to the receptor and improving insulin signaling we identified a chemical probe, C8, that reduced IR-Grb14 interaction, stimulated the MAP kinase pathway and induced the production of PIP_3_ by PI3-kinase. Importantly, studies on primary hepatocytes showed that C8 increased the effect of insulin on the expression of its target genes. It should be underlined that an increased lipogenesis induced by C8 may lead to the accumulation of triglycerides in liver cells and cause hepatic steatosis, which could be a problem in the context of diabetes therapy. However, hepatic steatosis is not necessarily associated with the development of insulin resistance, as illustrated by an increasing number of studies^38^. More particularly, increased levels of monounsaturated fatty acids newly synthesized through the *de novo* lipogenic pathway can exert a protective effect on liver insulin sensitivity^39^. It would thus be interesting to analyze the effects of C8 on the potential development of hepatic steatosis and on the composition of liver lipid stores in mice.

Protein-protein interactions play a central role in a variety of biological processes and structures. The inhibition of protein-protein interaction by small molecules has long been thought to be difficult to achieve, mostly due to the large and relatively flat and featureless interfacial areas involved. However, the development of protein-protein interaction inhibitors offers attractive opportunities for novel therapeutic intervention and some successes have recently emerged in the field of inflammation (Jak/Stat, IL-2R), apoptosis (Bcl-2, p53) or virology (HIV-1 protease)^40–44^. Inhibition of IR-Grb14 may be an additional example of the potential of this strategy for the development of small molecules of therapeutic interest for the treatment of important human pathologies.

## Methods

All methods were carried out in accordance with relevant guidelines and regulations.

### Virtual docking and scoring

The crystallographic structure of the activated IRTK in complex with the Grb14 BPS domain^15^ (PDB ID: 2AUH, resolution 3.2 Å) was used for *in silico* screening. A multi-step docking/scoring protocol employing MS-DOCK^45^ and Surflex^46^ was used. Two binding pockets could be identified at the surface of the IR and the compound collection was docked on these two different pockets. The ChemBridge database (the starting collection contained about 500 000 unique molecules) was filtered with our software FAF-Drugs^47^ and an in-house “soft” drug-like physicochemical property filter (http://fafdrugs4.mti.univ-paris-diderot.fr/filters.html) resulting in a final collection of 340,000 molecules. The 3D generation was performed with our in-house program DG-AMMOS^48^.

### Chemicals

Sulfafurazole (compound B) and all other chemical reagents and solvents were obtained commercially (Sigma-Aldrich). (*E)*-3-(5-methyl-2-furanyl)-2-propenoic acid (compound A) was prepared from 5-methyl-2-furfuraldehyde according to a published procedure^49^.

#### Synthesis of C8

In a 10 ml glass vessel, a solution of compound A (76 mg; 0.5 mmol) in dry dimethylformamide (625 μl) under argon at 0 °C was treated with 1-bis(dimethylamino)methylene-1*H*-1,2,3-triazolo[4,5-*b*]pyridinium 3-oxide hexafluoro-phosphate (HATU) (190 mg; 0.5 mmol) followed by *N*,*N*-diisopropylethylamine (87 μl; 0.5 mmol). The mixture was stirred for 5 min at 0 °C, then sulfafurazole (134 mg; 0.5 mmol) was added. The mixture was stirred at room temperature for 15 min and then irradiated (70 °C; 40 W) for 25 min in a microwave reactor (CEM Discover). The mixture was digested with ethyl acetate (100 ml) and extracted with a saturated aqueous NaHCO_3_ solution (50 ml). The aqueous extract was treated with 6 M HCl (6 ml) so that acidic pH was obtained and a precipitate appeared. The precipitate was isolated by Buchner filtration, rinsed with distilled water (2 × 5 ml) and dried at 40 °C for 6 h to provide C8 (89 mg; 44%) as a yellow solid; mp = 150–151 °C; *R*_*f*_ = 0.33 (EtOAc); IR (ATR) ν: 3315, 3112, 3060, 2839, 2774, 1673, 1625, 1589, 1540, 1526, 1497, 1419, 1368, 1334, 1348, 1253, 1189, 1162 cm^−1^; ^1^H NMR (250 MHz, DMSO-*d*_6_) *δ*: 10.95 (1H, bs), 10.60 (1H, s), 7.88 (2H, d, *J* = 9.0 Hz), 7.72 (2H, d, *J* = 9.0 Hz), 7.37 (1H, d, *J* = 15.5 Hz), 6.80 (1H, d, *J* = 3.3 Hz), 6.37 (1H, d, *J* = 15.5 Hz), 6.28 (1H, d, *J* = 3.3 Hz), 2.35 (3H, s), 2.10 (3H, s), 1.63 (3H, s); ^13^C NMR (75 MHz, DMSO-*d*_6_) *δ*: 164.2, 161.4, 155.6, 154.9, 149.4, 143.7, 133.5, 128.2, 128.0, 118.8, 117.1, 116.9, 109.2, 105.1, 13.5, 10.3, 5.8; HRMS: *m/z* found: 424.0925 [M + Na]^+^; C_19_H_19_N_3_NaO_5_S requires 424.0938.

### Cell culture

HEK-293T cells were maintained in Dulbecco’s modified Eagle’s medium containing 4.5 g/l glucose and 10% fetal bovine serum (FBS). Cells were transfected using FuGENE 6 (Promega) according to the manufacturer’s instructions. αML12 cells were cultured in DMEM/Ham’s F12 medium containing 10% FBS and supplemented with 5 µg/ml insulin, 5 µg/ml transferrin, 5 µg/ml selenium, 40 ng/ml dexamethasone, 100 units/ml penicillin and 100 µg/ml streptomycin. Cells were incubated in serum free medium and stimulated with or without 5 nM insulin and 50 µM of compound C8 for 4 h at 37 °C. Cells were incubated in serum free medium and stimulated with or without 100 nM insulin and 25 µM of compound C1-13 for 4 h at 37 °C.

Mouse primary hepatocytes were isolated as described previously^50^. Cells were cultured in Medium 199. For lipogenic gene expression analysis, cells were cultured for 24 h in the presence of 5 mM glucose or 25 mM glucose/10 nM insulin in the absence or the presence of 50 µM of compound C8. For gluconeogenic gene expression analysis, cells were preincubated for 1 h in the presence of 5 mM glucose with or without 10 nM glucagon and then treated for 8 h in the absence or the presence of 1 nM insulin and 50 µM of compound C8.

### Expression vectors

IR, IR-Rluc, Grb14-YFP, YFP-PTP1B, YFP-targeted to the plasma membrane and Luc-Akt-PH expression vectors have been described previously^6,27,33,51^. The cDNA coding for Raf-RBD-Luc was obtained by fusing the Ras Binding Domain (RBD) of Raf to the N-terminus of *Renilla* luciferase. YFP-Ras plasmid was provided by Van der Meer W.R., University of Leiden. YFP-Grb10 plasmid was provided by Liu F., University of Texas. The cDNA coding for IRTK48-Rluc was obtained by fusing the tyrosine kinase domain of the insulin receptor to the N-terminus of *Renilla* luciferase.

### A-cellular BRET assays

#### Study of the IR-Grb14 interaction using an *in vitro* BRET assay

HEK-293T cells were transfected with 6 μg of IR-Rluc cDNA, 6 μg of Grb14-YFP or 6 μg of empty vector cDNAs per 100 mm dish. Two days after transfection, cells transfected with Grb14-YFP or empty vector were lysed, cleared and extracts were aliquoted and stored at −80 °C for subsequent use. Cells transfected with IR-RLuc were stimulated or not with 100 nM of insulin during 10 minutes before lysis. Fusion receptors were partially purified by chromatography on wheat-germ lectin Sepharose (WGL) as described previously^32^. After elution with N-acetylglucosamine (0.5 M), fractions enriched in Rluc activity were pooled, aliquoted and stored at −80 °C for subsequent use.

A-cellular BRET assay was performed using 20 µl of IR-Rluc eluate preincubated in 96-well microplates for 20 min at room temperature with different concentrations of the compounds to be tested, or DMSO as control. 20 µl of extract obtained from Grb14-YFP transfected cells or control extract (from empty vector transfected cells) were added, and after 40 min of incubation, the substrate of luciferase cœlenterazine (Interchim, Montluçon, France) was added (final concentration of 2.5 μM). Light emission acquisition was then performed at 480 and 530 nm. The BRET signal was expressed in milli-BRET units, corresponding to the BRET ratio multiplied by 1000. The BRET ratio was previously defined as the emission ratio 530/480 nm obtained when the two partners are coexpressed, corrected by the ratio 530/480 nm obtained for the luciferase-fused protein expressed alone^32,52^.

### Ligand-induced conformational changes within the insulin receptor

HEK-293T cells were transfected with 2.5 μg of IR-Rluc cDNA or with 1 μg of IR-Rluc and 1.5 μg of IR-EYFP cDNAs per 12-well plate. 48 h after transfection, cells were extracted in buffer containing 1% (w/v) Triton X-100, 20 mM MOPS, 2.5 mM benzamidine, 1 mM EDTA, 1 mM 4-(2-aminoethyl)benzenesulfonyl fluoride hydrochloride, and 1 μg/ml each aprotinin, pepstatin, antipain, and leupeptin. Fusion receptors were partially purified by chromatography on WGL as described above. *In vitro* measurement of BRET signal was performed using 5–10 μl of WGL eluate (approximately 2 μg of proteins) preincubated in 96-well microplates for 30 min at room temperature in a total volume of 55 μl containing 30 mM MOPS, 1 mM Na_3_VO_4_, and different concentrations of ligands. After preincubation, coelenterazine was added (final concentration of 4.5 μM). Light emission acquisition (at 480 and 530 nm) was then started.

### BRET in living cells

HEK-293T cells were seeded at a density of 1.5 × 10^5^ cells per 12 well plate and transfected 24 h later using FuGene 6 and the following combinations of vectors: i) Raf-RBD-Luc (400 ng), IR (400 ng) and either empty vector or WT or mutated pEYFP-RasN17 (400 ng); ii) Luc-Akt-PH (700 ng) and either empty vector or YFP-mem (YFP-fused to a membrane targeting sequence^33^, 300 ng). BRET measurements on intact cells were performed essentially as described previously^33^. Briefly, the day after transfection, cells were detached with Trypsin-EDTA (Invitrogen) and resuspended in Dulbecco’s modified Eagle’s medium supplemented with 4.5 g/l glucose and 10% fetal bovine serum. Cells were then transferred into 96-well microplates at a density of 30,000 cells per well. Twenty four hours later, cells were incubated in PBS at room temperature for 10 min with cœlenterazine at a final concentration of 2.5 μM. Cells were treated in the absence or the presence of different concentrations of ligands (insulin and test compounds), and light-emission acquisition was then started. The interaction was monitored for at least 30 min using the Mithras LB 940. The BRET signals were expressed as described above.

### Western blotting and co-Immunoprecipitation

For protein analysis, cells were lysed on ice for 15 min in 200 µl lysis buffer/well containing 50 mM Tris-HCl (pH 8), 150 mM NaCl, 1% Triton-X-100, 50 mM NaF, 10 mM β-Glycerophosphate, 1 mM Orthovanadate and a protease inhibitor cocktail (AEBSF 24 µg/ml, Pepstatine, Aprotinine, Leupeptine, and Antipain 1 µg/ml each) and extracts were cleared by a 15,000 × g centrifugation at 4 °C for 15 min^53^. Cell lysates were analyzed using 8% SDS–PAGE gels and immunodetected with the following antibodies: anti-GFP (Roche Diagnostics), anti-Luc (Chemicon), anti-IR (C-19, Santa Cruz Biotechnology), anti-4G10 (Santa Cruz Biotechnology), anti-3PY-IR (Biosource), anti-pAkt (Ser473, Cell Signaling), anti-Akt1/2/3 (H-136, Santa Cruz Biotechnology).

For co-immunoprecipitation assays of IR with Grb14, Wheat Germ Lectin (WGL)–partially purified receptors prepared from control or insulin-stimulated cells were preincubated for 20 minutes with control DMSO or with different concentrations of compounds, and the Grb14-YFP protein extract was added for 40 min. Protein extracts were then incubated for 1 h at room temperature with 5 μl anti-GFP antibody in the presence of protein G-agarose beads. The beads were washed three times in lysis buffer and the resulting immunoprecipitates were submitted to SDS-PAGE analysis and immunodetected with the indicated antibodies. Quantifications of Western blots represent the signal corresponding to immunoprecipitated Grb14 adjusted to the signal corresponding to immunoprecipitated IRβ.

For co-immunoprecipitation assays of IRTK with Grb14 or Grb10, HEK-293T cells were transfected in 6-well plates using FuGENE 6 with 600 ng of IRTK48-Rluc plasmid and 600 ng of Grb14-YFP or YFP-Grb10 plasmid. Two days after transfection, cells were stimulated with 50 µM of compound C8 for 4 h. Cells were lysed as described above and protein and GFP contents of the lysates were quantified respectively using the BCA Protein Quantification Kit and the Fusion microplate analyzer. About 500 μg of proteins were incubated for 2h30 min at 4 °C with 5 μl of anti-GFP antibody in the presence of protein G-agarose beads. The beads were washed and bound proteins were analyzed as described above.

### Insulin receptor autophosphorylation

Partially purified IR-Rluc fusions (5 μl) were preincubated for 30 min at room temperature in a total volume of 60 μl containing 20 mM Tris (pH 7.4), 12 mM MgCl2, 2 mM MnCl2, and different concentrations of ligands (5 or 10 nM insulin and 50 µM of compound C8). The autophosphorylation reaction was initiated by adding 5 μl of ATP (final concentration, 100 μM) for 10 min and stopped by the addition of SDS-polyacrylamide gel electrophoresis sample buffer^32^. Autophosphorylation of the receptors was assessed by immunoblotting using the 4G10 antiphosphotyrosine antibody.

For autophosphorylation of endogenous insulin receptors in intact cells, HEK-293T cells were incubated for 5 min in the absence or the presence of 5 nM insulin and 50 µM of compound C8 and then lysed as described above. Clarified extracts (500 µg of proteins) were immunoprecipitated with anti-IR antibody and protein G-sepharose beads for 2 h 30 min at 4 °C^54^. After 3 washes, proteins were eluted from the beads in Laemmli buffer. Autophosphorylation of the endogenous receptors was assessed by immunoblotting using the anti-3PY-IR antibody.

### Isolation of total mRNA and analysis by RT-qPCR

Total cellular RNA was extracted from cultured hepatocytes using the TRIZOL reagent and reverse-transcribed as described previously^55^. Quantitative PCR was performed using a Lightcycler system and SYBR Green detection of amplified products (Roche Applied Science) using the following primer sequences, and gene expression was normalized over 18S RNA levels: steroid responsive element binding protein-1c (SREBP-1c), forward 5′-GACCCTTCCAGGAAACACTC-3′, reverse 5′-TGTTTGTTCTAGGGGCTGC-3′; acetyl CoA carboxylase (ACC), forward 5′-GAGGTGGCTAAGAGGAGGCTCT-3′; fatty acid synthase (FAS), forward 5′- TTCCAAGACGAAAATGATGC-3′, reverse 5′-AATTGTGGGATCAGGAGAGC-3′; steroyl CoA desaturase-1 (SCD1), forward 5′-CCGGAGACCCTTAGATCGA-3′, reverse 5′- TAGCCTGTAAAAGATTTCTGCAAA-3′; phosphoenolpyruvate carboxykinase (PEPCK), forward 5′-TGGCTACGTCCCTAAGGAA-3′, reverse 5′-GGTCCTCCAGATACTTGTCGA-3′; glucose-6-phosphatase (G6Pase), forward 5′-TTACCAGCCTCCTGTCGG-3′, reverse 5′- GACACAACTGAAGCCGGTTAG-3′; 18S, forward 5′- CCATCCAATCGGTAGTAGCG-3′, reverse 5′- GTAACCCGTTGAACCCCATT-3′.

### Cell Viability Assay

Cell viability was assessed after 72 h of culture using the Uptiblue assay (Interchim) according to the manufacturer’s instructions. Cells were seeded in 96-well plates (1 or 1,5 × 10^4^ cells/well). Three replicate wells were used for each experimental condition.

### Equipment and settings

Blots from Figs 1–6 were obtained by autoradiography using GE Healthcare Amersham Hyperfilm^TM^ ECL. Western blots were scanned using Epson Perfection 1670 Scanner and quantified using an image processor software (ImageJ). Blots from Fig. 7 and Supplementary Figs 4–5 were obtained using the Odyssey Infrared Imaging System (LI-COR Biosciences). BRET experiments were performed using the Mithras LB 940 (Berthold technologies). Uptiblue measurements were performed using a Typhoon^TM^ FLA 9000 (GE Healthcare).

### Statistical analysis

Statistical analyses were performed with Prism software (GraphPad) using either a *t* test or a one-way analysis of variance (ANOVA) followed by appropriated post-test as indicated in figure legends.

## Electronic supplementary material

Supplementary dataset 1
