# Supplementary material for: Identification of insulin-sensitizing molecules acting by disrupting the interaction between the Insulin Receptor and Grb14
Source: Sci Rep. 2017 Dec 4;7:16901. doi: 10.1038/s41598-017-17122-6 (PMC5715071; doi:10.1038/s41598-017-17122-6)
Supplement: Supplementary file 1 [file 41598_2017_17122_MOESM1_ESM.doc]

# Identification of insulin-sensitizing molecules acting by disrupting the interaction between the Insulin Receptor and Grb14

Anaïs Gondoin, Cornélia Hampe, Richard Eudes, Cyril Fayolle, Cécile Pierre-Eugène, Maria Miteva, Bruno O. Villoutreix, Florence Charnay-Pouget, David J. Aitken, Tarik Issad and Anne-Francoise Burnol

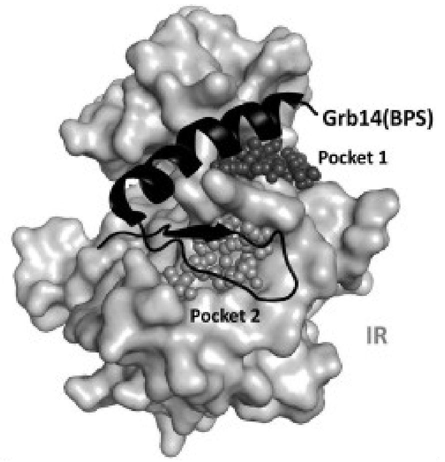


**Supplementary Figure 1:**
Crystal structure of the complex between Grb14 (BPS) region (ribbon diagram in black) and the IR (solid surface in grey). Two binding pockets (1 and 2) were screened *in silico* in order to search for chemical probes that could impede the protein-protein interaction.

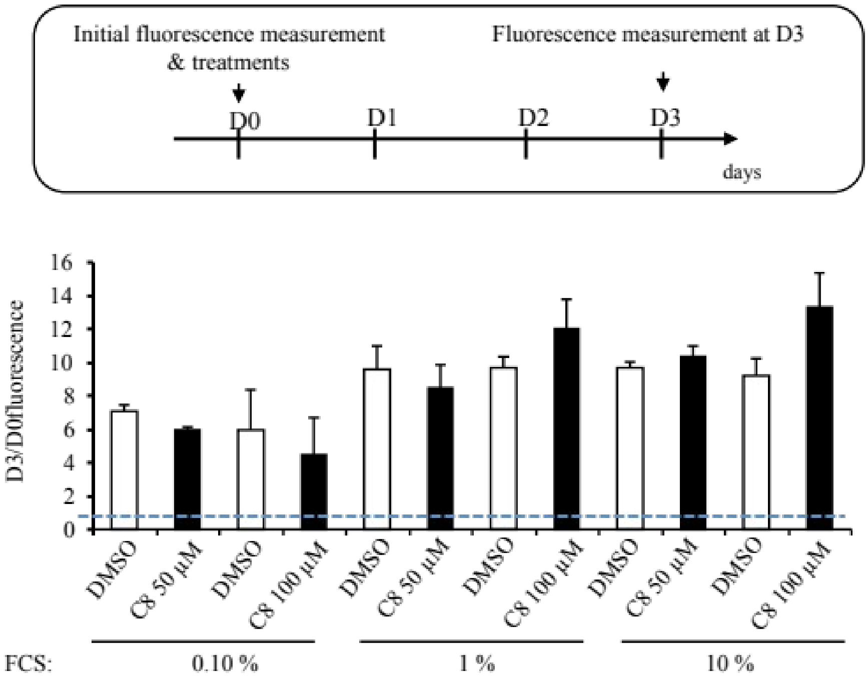


**Supplementary Figure 2: Evaluation of C8 toxicity**

Effect of C8 on cell growth and survival in HEK-293T cells after 72 h of culture using the UptiBlue assay. The histograms represent the rate of cells growth obtained by dividing the fluorescence at 72 h (D3) by the initial fluorescence (D0). The results are the mean ± SEM of 3 independent experiments performed in triplicate. FCS: fetal calf serum.

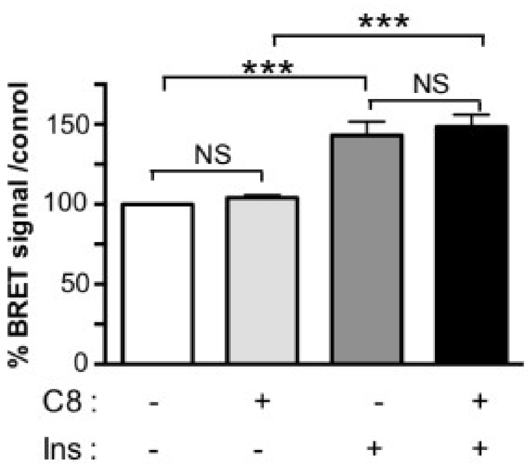


**Supplementary Figure 3: Effect of C8 on IR-PTP1B interaction**

IR-PTP1B interaction was measured by BRET assays in HEK293T cells. Cells were transfected with IR-luc and YFP-PTP1B expression plasmids, and cells were treated or not with 5 nM insulin and 25µM of C8 as indicated. Results, expressed as percent increase of BRET signal obtained in control cells, are the mean ± SEM of 5 independent experiments (*** $p<0.001$ using ANOVA followed by Tukey test).

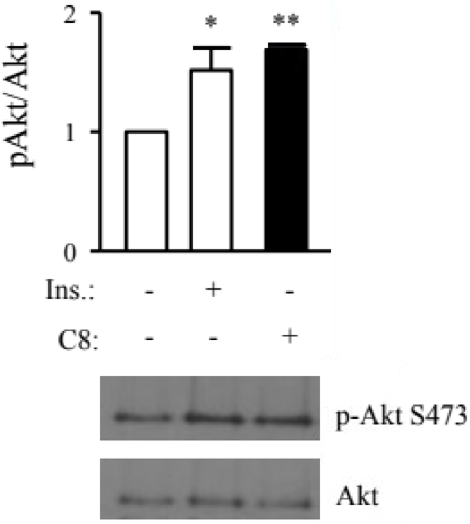


**Supplementary Figure 4: Effect of C8 on Akt phosphorylation in αML12 cells**
αML12 cells were stimulated with 5 nM insulin or 50 µM of compound C8 for 4 h. Phosphorylation of Akt was analyzed by Western blotting. Histograms represent the means ± SEM of the densitometric quantification of 4 independent experiments. (* $p<0.05$, ** $p<0.01$ effect compared with basal, using ANOVA followed by Dunnett Multiple Comparison Test).

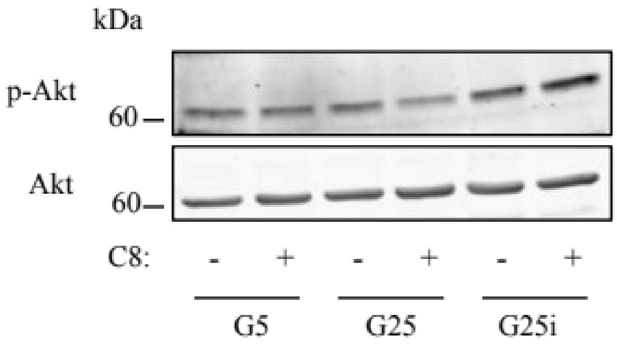


**Supplementary Figure 5: Effect of C8 on Akt phosphorylation in primary mouse hepatocytes.** Uncropped blots corresponding to Figure 7. Primary mouse hepatocytes were cultured for 24 h in 5 mM glucose (G5) or 25 mM glucose in the absence (G25) or in the presence of 10 nM insulin (G25i), with or without the addition of 50 µM of the compound G8, as indicated. Cell lysates were analyzed by Western blotting using the indicated antibodies.
